# ROR1 expression as a biomarker for predicting prognosis in patients with colorectal cancer

**DOI:** 10.18632/oncotarget.15860

**Published:** 2017-03-02

**Authors:** Jian-Kang Zhou, Yu-Zhu Zheng, Xue-Sha Liu, Qiheng Gou, Rui Ma, Cheng-Lin Guo, Carlo M. Croce, Lunxu Liu, Yong Peng

**Affiliations:** ^1^ Department of Thoracic Surgery and Lab of Non-coding RNAs in Diseases, State Key Laboratory of Biotherapy, West China Hospital, Sichuan University and Collaborative Innovation Center for Biotherapy, Chengdu, Sichuan, China; ^2^ Department of Thoracic Oncology, Cancer Center, West China Hospital, Sichuan University, Chengdu, Sichuan, China; ^3^ Department of Oncology, The Third People's Hospital of Chengdu, Chengdu, Sichuan, China; ^4^ Department of Cancer Biology and Genetics, The Ohio State University, Columbus, OH, USA

**Keywords:** receptor-tyrosine-kinase-like orphan receptor 1, colorectal cancer, prognostic factor, tissue microarray, immunohistochemistry

## Abstract

There is a lack of reliable prognosis biomarker in the current treatment of colorectal cancer. The receptor-tyrosine-kinase-like orphan receptor 1 (ROR1) is overexpressed and associated with poor prognosis in certain tumors. This study aimed to explore the prognostic significance of ROR1 in colorectal cancer. Western blot analysis and immunohistochemistry showed that the expression of ROR1 in colorectal cancer was significantly higher than that in the adjacent normal tissues. ROR1 expression was positively associated with the clinical stage and lymph-node metastasis (*p* < 0.01). Kaplan-Meier survival analysis revealed that patients with higher ROR1 expression had a significantly shorter overall survival (*p* < 0.01). Multivariate Cox regression analysis confirmed that ROR1 is an independent prognostic marker in colorectal cancer (p = 0.002, HR = 2.08, 95% CI: 1.314–3.292). Thus, our study demonstrated that ROR1 expression is correlated with malignant attributes and may serve as a novel prognostic marker and therapeutic target for colorectal cancer.

## INTRODUCTION

Colorectal cancer (CRC) accounts for 8% of all cancer-related deaths [1]. Usually, localized CRC patients (stage I-II) are curable by surgery or radical radiotherapy, while patients at stage III-IV need systematic therapy including chemotherapy and target therapy to improve survival and minimize the possibility of relapse [2]. However, lack of accurate biomarkers to identify early-stage and low-risk patients often leads to overtreatment [3]. Therefore, it is of critical importance to identify diagnostic and prognostic biomarkers to improve CRC patients' survival.

Currently, the standard for determining the prognosis and establishing clinical treatment program of CRC patients is the American Joint Committee on Cancer (AJCC) staging system [4]. However, the pathological staging is not accurate enough to predict the recurrence. About 10-20% of stage II patients and 30-40% of stage III patients still develop tumor recurrence [5]. Some new biomarkers are under development to make supplement for prognosis and treatment. For example, microsatellite instability (MSI) is identified as a prognostic factor for early stage CRC patients [6], and KRAS is found to be a predictive marker in EGFR-targeted therapy of advanced CRC [7]. Therefore, identification of novel and specific biomarkers with clinicopathological and prognostic significance is vital for CRC management.

ROR1, a member of the ROR receptor tyrosine kinase family and a transmembrane glycoprotein, plays a pivotal role in differentiation, proliferation and survival. ROR1 is primarily expressed during embryonic and fetal development, whereas it is absent in most mature tissues [8, 9]. Recently, ROR1 expression was reported to elevate in human leukemia and a variety of solid malignancies [10–14], suggesting that ROR1 may serve as a potential target for cancer therapy. Furthermore, higher expression of ROR1 is associated with more aggressive and poorer prognosis in breast, ovarian and lung cancers, in which ROR1 regulates expression of genes involved in epithelial-mesenchymal transition (EMT) [15]. These findings nurtured our assumption that there is a close relationship between ROR1 expression and clinical parameters of CRC patients, while the potential of ROR1 as a target for CRC therapy needs further investigation.

In this study, we explored the diagnostic and prognostic significance of ROR1 as a potential biomarker for CRC. Firstly, we examined ROR1 expression in CRC tissues by Western blot and in CRC cells by flow cytometry. Then we systematically analyzed ROR1 expression in CRC patients on tissue microarray using immunohistochemistry (IHC). Finally, we evaluated the correlation of ROR1 expression with clinicopathological characteristics as well as survival of CRC patients.

## RESULTS

### ROR1 expression increased in CRC samples

**Figure 1:**
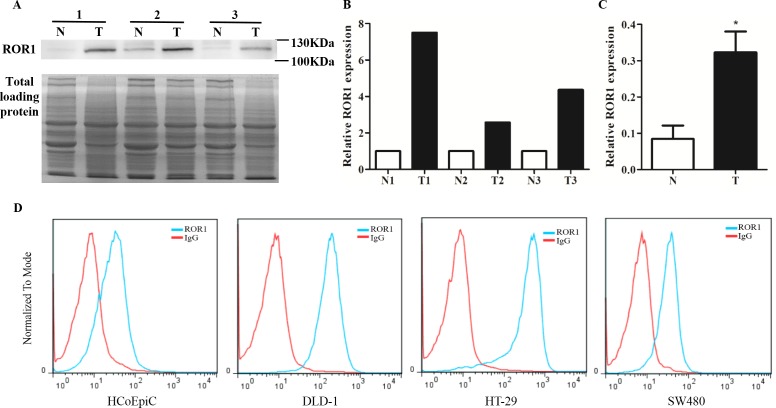
ROR1 expression in CRC tissues and CRC cells **(A)** Western blot of ROR1 expression in human CRC tissues (T) and their adjacent normal tissues (N). Total proteins were used as loading control. **(B, C)** The intensity of each lane was calculated by Image J software and analyzed by paired t-test. Statistics results showed that *P*<0.001. **(D)** Flow cytometry of ROR1 expression in the immortalized normal colon cells and CRC cells.

Western blot was employed to determine ROR1 expression in CRC tissues and their adjacent normal tissues. As shown in Figure 1A, CRC tissues exhibited high expression of ROR1 protein, whereas their matched normal tissues expressed little ROR1 protein. Total proteins stained by Coomassie blue were used as control, and the intensity of each lane was calculated by Image J software. The quantification data showed that ROR1 expression in CRC tissue was significantly higher than that in normal tissue (*p* < 0.001) (Figure 1B and 1C). We also examined ROR1 expression in colorectal cancer cells by flow cytometry. Although there was no change in ROR1 level observed in SW480 cells, the expression of ROR1 protein significantly increased in other two investigated CRC cells, DLD-1 and HT-29, when compared with immortalized normal colon cell HCoEpiC (Figure 1D), which implied that ROR1 expression widely elevates in the CRC cells.

### Clinical parameters of CRC patients

To further validate overexpression of ROR1 in CRC patients, we performed immunohistochemistry staining to analyze ROR1 expression on the tissue microarray containing 186 CRC cases. The main clinicopathological parameters of these patients are summarized in Table 1. Overall, 76 female patients and 109 male patients, ranging in age from 30 years to 91 years (mean age of 65.7 years), were included in this study. According to the 7^th^ edition of AJCC TNM staging system, there were 106 patients (57%) at stages I and II, and 80 patients (43%) at stages III and IV. Based on the World Health Organization (WHO) pathological grade system, 140 patients (75.3%) were at grade I-II and 46 patients (24.7%) were at grade III. Among these patients, 79 patients (42.5%) showed positive lymph node metastasis, whereas 107 patients (57.5%) showed negative lymph node metastasis. The overall follow-up durations of these patients ranged from 1 to 87 months (mean time 56 months). There were 99 patients alive at the end of the follow-up and the overall survival (OS) rate was 53.2% in this study.

**Table 1:** The clinical parameters of 186 CRC patients

Clinical Features	N=186
Age	
≤65	83(44.7)
>65	102(54.8)
Unknown	1(0.5)
Gender	
Female	76(40.9)
Male	109(58.6)
Unknown	1(0.5)
Pathological grade	
I-II	140 (75.3)
III	46 (24.7)
AJCC7 stage*	
I-II	106(57)
III-IV	80(43)
Lymph node metastasis	
Positive	79(42.5)
Negative	107(57.5)
Vital status	
Alive	99(53.2)
Death	87(46.8)

Data are presented as No. (%).

*Each case was reassigned for pathological stage according to the 7th edition of AJCC.

### Immunohistochemistry analysis of ROR1 expression in CRC tissues

**Figure 2:**
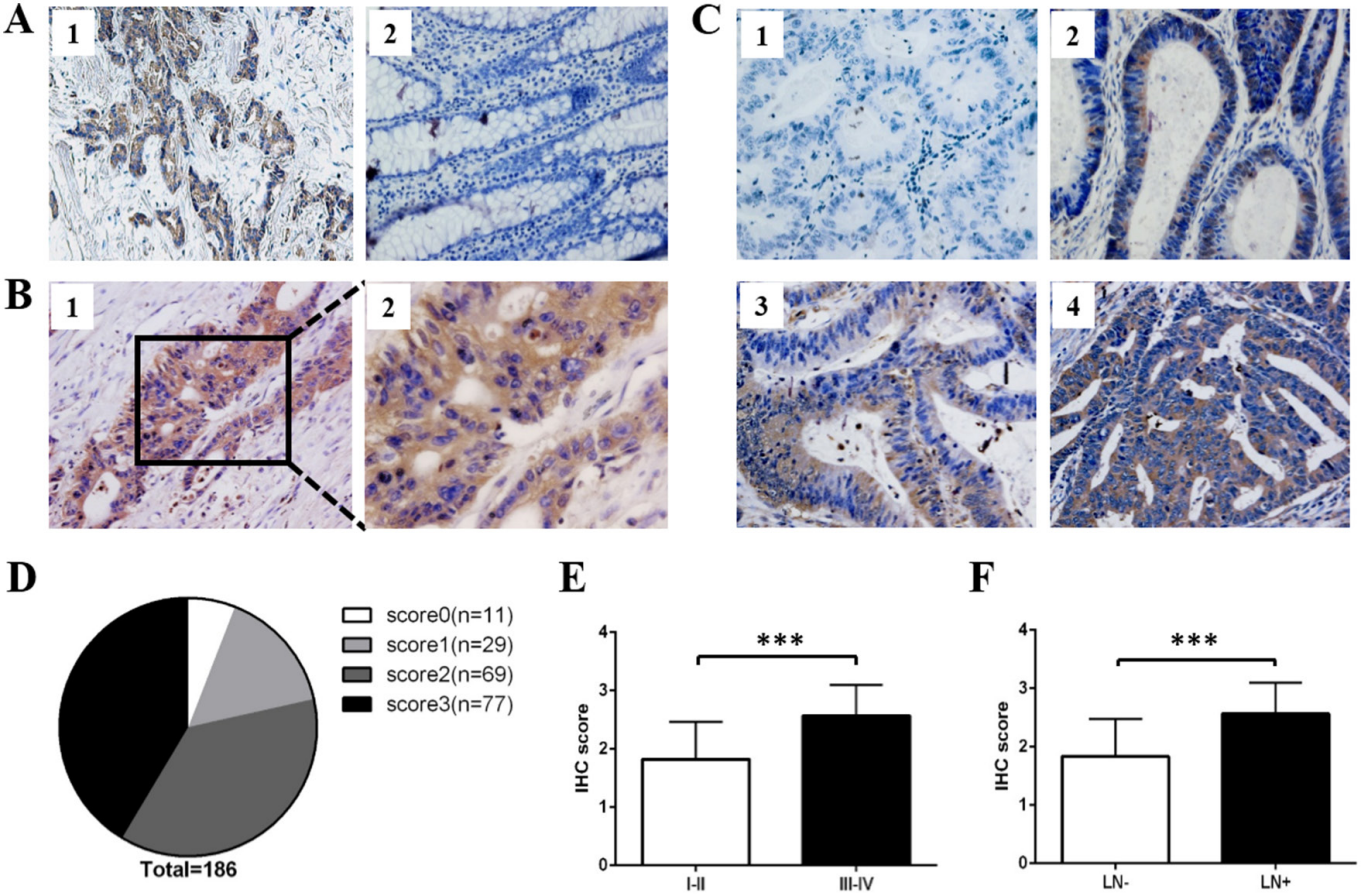
ROR1 expression on CRC tissue microarray by immunohistochemistry staining **(A)** The IHC staining of ROR1 expression in breast tumor (1) and its adjacent normal tissue (2). **(B)** Detection of ROR1 expression in CRC tissues. Positive ROR1 staining was shown in brown color and the nucleus counterstained with hematoxylin was shown in blue color. The magnification was ×200 in B1, ×400 in B2. **(C)** Different levels of ROR1 expression detected by TMA-IHC analysis. (1) Score 0 indicates that none or little cells exhibit ROR1 expression; (2) score 1 indicates that more than 25% of tumor cells exhibit weak ROR1 expression; (3) score 2 indicates more than 50% of tumor cells have weak expression or more than 25% of tumor cells have moderate ROR1 expression; (4) score 3 indicates more than 75% of tumor cells have moderate expression or more than 50% of tumor cells have strong ROR1 expression. **(D)** The proportion of negative (score 0), weak (score 1), moderate (score 2) and strong (score 3) staining for ROR1 protein in CRC patients. **(E)** The scores of ROR1 in CRC tissues at different stages were analyzed by two tail t-test (*P* < 0.001). **(F)** The scores of ROR1 in CRC tissues with different status of lymph node metastasis were analyzed by two tail t-test (*P* < 0.001). LN, lymph node.

The breast tumors were reported to express high level of ROR1 when compared with their adjacent normal tissues [13]. Therefore, we used breast tumors as positive controls to verify the specificity of ROR1 antibody. As expected, higher ROR1 expression was shown in the breast tumor tissue compared to normal tissue (Figure 2A), validating that the antibody we used can specifically detect ROR1 expression. Then we analyzed ROR1 expression in CRC patients. Overall, ROR1 expression was found in the primary lesion of CRC tissue, but not observed in the adjacent normal tissue (Figure 2B). We set up the scoring standard (Figure 2C), and two independent researchers scored them to minimize the bias of IHC scoring. As shown in Figure 2D, the positive ROR1 expression rate in CRC patients was 94.09% (175/186), among which 16.6% (29/175) exhibited weak expression (score 1), 39.4% (69/175) moderate expression (score 2) and 44% (77/175) strong expression (score 3). The mean score of all CRC tissues was 2.14. Therefore, we divided CRC patients into two groups as follows: score ≤ 2 belong to low ROR1 expression group and score > 2 belongs to high ROR1 expression group. Moreover, our results showed that higher score of ROR1 was positively correlated to advanced tumor stage (Figure 2E) and positive lymph node metastasis (Figure 2F) in CRC patients.

### Correlation of ROR1 expression with clinicopathological characteristics in CRC patients

To evaluate ROR1 function during CRC progression, the correlation of ROR1 expression with clinicopathological characteristics of CRC patients was analyzed by Chi-square test. As shown in Table 2, high ROR1 expression in CRC patients was positively associated with poor pathological grading (*p* = 0.013), advanced-stages (stage III and IV) (*p* < 0.001) and positive lymph node metastasis (*p* < 0.001). There was no significant correlation between ROR1 expression and other clinical parameters such as age, gender, tumor diameter and histological type.

**Table 2:** Correlation between ROR1 expression and the clinicopathologic parameters in CRC patients

Clinicopathologic variables	n	ROR1		χ2	*p* - value
		Low	High		
All cases	186	82	104		
Age					
≤65	83	36	47	0.055	0.814
>65	102	46	56		
Unknown	1	0	1		
Gender					
Female	76	32	44	0.257	0.612
Male	109	50	59		
Unknown	1	0	1		
Pathological grading				6.209	0.013*
I-II	140	69	71		
III	46	13	33		
Stage				56.815	<0.001*
I-II	106	72	34		
III-IV	80	10	70		
Lymph node metastasis				55.025	<0.001*
Positive	79	10	69		
Negative	107	72	35		

* *p* < 0.05.

### Survival analysis

**Figure 3:**
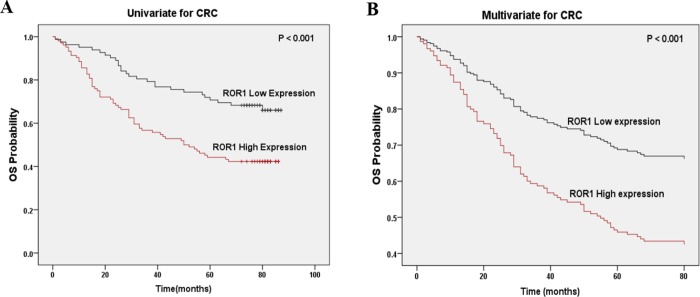
Correlation of ROR1 expression with overall survival in CRC patients **(A)** Kaplan-Meier survival analysis of ROR1 expression in CRC patients. The low ROR1 expression group had longer OS than the high ROR1 expression group. **(B)** Multivariate Cox regression survival analysis in CRC patients. ROR1 expression could serve as an independent prognostic factor.

A total of 166 patients were included in the survival analysis. To evaluate if there is correlation between ROR1 expression and survival, the Cox proportional hazards model was performed. According to the univariate analysis, the OS time of CRC patients was associated with ROR1 expression (*p* = 0.001), the 7^th^ edition of AJCC TNM stages (*p* = 0.001), pathological grading (*p* = 0.002) and positive lymph node metastasis (*p* = 0.006) (Table 3). Kaplan-Meier survival curves further confirmed that higher ROR1 expression was related to shorter OS time (Figure 3A). Meanwhile, the multivariate analysis indicated that ROR1 could serve as an independent prognostic factor in CRC patients (HR = 2.08, *p* = 0.002) (Figure 3B and Table 3).

**Table 3:** Univariate and multivariate analysis of prognostic factors in CRC for overall survival

	Univariate analysis			Multivariate analysis		
	HR	p>│z│	95% CI	HR	p>│z│	95% CI
ROR1 expression	2.224	0.001*	1.411-3.507	2.08	0.002*	1.314-3.292
High vs Low						
Age	1.546	0.052	0.996-2.398			
>64 years vs ≤64 years						
Gender	0.781	0.263	0.506-1.204			
Female vs Male						
AJCC7 Stage	1.869	0.001*	1.226-2.849			
Stage I and II vs Stage III and IV						
Pathological Grading	2.027	0.002*	1.297-3.169	1.832	0.008*	1.167-2.876
Grade I and II vs Grade III						
Lymph node metastasis	1.799	0.006*	1.181-2.741			
Positive vs Negative						

* *p* < 0.05

## DISCUSSION

Although great improvements have been made in diagnostic and therapeutic technologies for CRC treatment in recent years, the 5-year OS rate for CRC patients remains low. Currently, the prognosis and treatment of CRC patients still depend on the pathological stage of tumors evaluated by AJCC TNM staging system [16]. Nevertheless, some studies have demonstrated that stage II patients have even worse survival data than those of stage III [17, 18], suggesting that AJCC TNM staging system is not sufficient and reliable enough. As a supplementary method, an increasing number of biomarkers, such as KRAS and BRAF, have been used in clinical practice [19]. But only a few CRC patients benefit from the treatment based on KARS and BRAF subtypes. Therefore, identifying a more reliable prognostic biomarker and therapeutic target is urgently needed.

As receptor tyrosine kinases (RTKs) activate various signaling pathways and regulate cellular proliferation, migration and angiogenesis, aberrant expression or activation of certain RTKs may contribute to tumorigenesis in multiple ways. The orphan receptor tyrosine kinase ROR1 is a transmembrane glycoprotein with high expression during embryonic and fetal development, while with little expression in adult tissues [9]. Recently, high ROR1 expression was also observed in acute lymphocytic leukemia [20], chronic lymphocytic leukemia [21], ovarian cancer [14, 22], gastric cancer [23], breast cancer [13] and lung cancer [10]. But there is a lack of exploration on ROR1 expression in CRC. In this study, we assessed ROR1 level in CRC tissues and found that ROR1 is highly expressed in CRC tissues. About 94% of investigated CRC patients showed positive ROR1 expression. However, the molecular mechanism of the tumor-specific ROR1 expression remains unclear and investigations are still ongoing. In lung adenocarcinoma cell lines NCI-H1975 and SK-LC-5, NKX2-1, a homeodomain transcription factor, has been shown to directly induce the expression of ROR1, which in turn sustains a favorable balance between prosurvival PI3K-AKT and pro-apoptotic p38 signaling [24]. In addition, we demonstrated that both DLD-1 and HT-29 CRC cells have high ROR1 expression, whereas SW480 cell doesn’t. Given that DLD-1 and HT-29 cells have PIK3CA gene mutation (E545K; D549N and P449T, respectively), and SW480 cell hasn't [25], activation of PI3K signaling caused by PIK3CA gene mutation may contribute to increased ROR1 expression. Therefore, we hypothesize that overexpression of NKX2-1 or activation of PI3K signaling contributes to maintaining high level of ROR1 in CRC patients, which, however, needs further in-depth study to verify in the future.

High ROR1 expression on cell membrane in human malignancies enables it to be a therapeutic target for both leukemia and some solid tumors. In a preclinical study, Cirmtuzumab (UC-961), a first-in-class humanized mAb against ROR1, exerted a good anti-tumor effect in CLL [26]. Several other high affinity ROR1 mAbs had been applied to CLL and MCL lymphoma [27]. Another therapeutic approach against ROR1 is CAR (Chimeric antigen receptor)-T cell therapy. Engineered T cells expressing a ROR1-specific CAR can recognize the tumor cells and exhibit significant anti-tumor effects in B-CLL [28, 29]. In this study, ROR1 expression was demonstrated to be significantly higher in CRC tissues than that in their adjacent normal tissues, suggesting that ROR1 could be a potential target for CRC therapy. TKIs and mAbs targeting ROR1 could be efficacious in CRC treatment. Patients with CRC might get more benefit from combination therapy of ROR1 target agents with traditional therapies.

Previous reports have indicated that high ROR1 expression was associated with metastasis in breast cancer and ovarian cancer [13, 30]. Mechanistically, ROR1 regulates expression of epithelial-mesenchymal transition (EMT) genes, such as SNAIL-1/2, E-cadherin, N-cadherin and vimentin [15, 31]. Silencing ROR1 expression in melanoma significantly inhibits cell migration and invasion through decreasing expression of vimentin and N-cadherin [32]. In our study, we also found a significant association between ROR1 expression and lymph node metastasis, which indicated that ROR1 may also be involved in the process of CRC metastasis. According to our survival analysis results, high expression of ROR1 protein was significantly associated with adverse OS in Univariate analysis and Kaplan-Meier curve. Multivariate analysis stated that ROR1 expression can be used as an independent prognostic factor in CRC patients. Therefore, ROR1 could serve as an independent biomarker to clinically predict the survival of CRC patients, while its functions and mechanisms in CRC need more investigation.

As a summary, we investigated the clinicopathological relevance of ROR1 expression in a large cohort of CRC patients. To our knowledge, this is the first report showing that ROR1 is highly expressed in CRC tissues when compared with their adjacent normal tissues. Moreover, ROR1 expression in CRC patients correlated with the 7th edition of AJCC TNM stage and lympho node metastasis status. The Kaplan-Meier curve indicated that the CRC patients with higher ROR1 expression had significantly shorter OS, and those with lower ROR1 expression had longer OS. Multivariate analysis further confirmed that ROR1 is an independent prognostic factor for CRC patients. In conclusion, ROR1 expression is correlated with malignant attributes of CRC and may serve as a novel prognostic biomarker and therapeutic target for CRC treatment.

## MATERIALS AND METHODS

### Patient tissue samples

Human colorectal cancer, breast cancer and their adjacent normal tissues were collected from West China Hospital, Sichuan University, with informed written consent from every patient. The tissue microarray slides containing 186 cases of colorectal cancer patients, and the corresponding clinicopathological information were obtained from Shanghai Outdo Biotech Co., Ltd. (SOBC). This study was approved by the ethics committee of the West China Hospital affiliated to Sichuan University, and all of the experiments were carried out in accordance with approved guidelines of Sichuan University.

### Western blot analysis

CRC tissues and their adjacent normal tissues were lysed in RIPA buffer with protease inhibitor cocktail. Total proteins, measured by BCA protein assay kit, were separated on 8% SDS-polyacrylamide gels and electrically transferred onto PVDF membrane (Millipore). Membrane was blocked for 1 h at room temperature prior to incubation with polyclonal rabbit anti-ROR1 antibody (Abcam, ab135669) at 4°C overnight. After washed with TBST, membrane was incubated in horseradish peroxidase-conjugated anti-rabbit antibody for 1 h, and developed by SuperSignal West Dura Extended Duration Substrate (Thermo Scientific).

### Flow cytometry analysis

Cells were collected and resuspended at a concentration of 1×10^7^/ml in PBS containing 2% FBS. Approximately 1×10^6^ cells were incubated on ice for 30 min with APC-conjugated anti-human ROR1 (Biolegend, cat#357806) or control IgG (Biolegend, cat#400120). After 3 times of wash, cell surface expression of ROR1 protein was detected by flow cytometry analysis on a FACSCalibur instrument (BD Biosciences). Data analysis was carried out using FlowJo software to compare ROR1 expression between normal cells and colorectal cancer cells.

### Immunohistochemical analysis

Paraffin tissue sections were deparaffinized in xylene and rehydrated gradually in serial dilutions of ethanol. Antigen retrieval was performed by autoclave treatment at 95°C for 3 min in 0.01M citrate buffer (pH = 6.0, ZSGB-BIO, Beijing). The slides were then incubated with 0.3% H_2_O_2_ for 10 min to quench endogenous peroxidase activity. After washed with PBS three times, the sections were blocked with goat serum for 15 min, followed by incubation with polyclonal rabbit anti-ROR1 antibody (1:20, Abcam, England) at 4°C overnight. After wash, sections were incubated with horseradish peroxidase-conjugated anti-rabbit antibody (ZSGB-BIO, Beijing) at 37°C for 15 min. Negative controls were obtained by replacing the primary antibody with PBS. The sections were stained with DAB^+^ substrate-chromogen solution (Maixin Biotech. Co., China). Counterstaining was performed with hematoxylin. ROR1 immunostaining was independently scored by two experienced individuals according to intensity and percentage of ROR1-positive cells. Staining intensity was scored as follows: 0 (negative), 1 (weakly positive), 2 (moderately positive), and 3 (strongly positive). The percentage of ROR1 positive cells was divided into 4 categories: 1 was for 0–10%, 2 for 11–50%, 3 for 51–80%, 4 for 81–100%.

### Statistical analysis

Statistical analyses were performed using SPSS 22.0 and GraphPad Prism 6 software. Briefly, Chi-square test was performed to analyze the association between ROR1 expression and clinicopathological features. Both Univariate and Multivariate Cox proportional hazards regression models were used to identify the independent prognostic factors of OS. Kaplan-Meier survival curves were constructed for survival analyses and differences were tested by the log-rank test. *p* < 0.05 was considered statistically significant.
